# A perspective on mental health literacy and mental health issues among Australian youth: Cultural, social, and environmental evidence!

**DOI:** 10.3389/fpubh.2023.1065784

**Published:** 2023-01-18

**Authors:** Hirukshi Bennett, Ben Allitt, Fahad Hanna

**Affiliations:** ^1^Public Health Program, Torrens University Australia, Melbourne, VIC, Australia; ^2^Higher Education College, Chisholm Institute, Dandenong, VIC, Australia

**Keywords:** mental health, mental health literacy (MHL), youth–young adults, culturally and linguistic diversity, social determinants, COVID-19, behavior, gender

## Abstract

Mental health literacy (MHL) helps improve mental health outcomes and reduce the impacts of mental illness. This study aims to reflect on scientific evidence on MHL levels, barriers to MHL, their impacts on mental health among Australian youth and interventions to overcome these barriers. The factors explored in the Perspective included; influence of social determinants, culturally and linguistically diverse (CALD) communities, help-seeking attitudes and behaviors. MHL intervention programs and MHL for improving mental health outcomes due to the recent COVID-19 pandemic were also explored. Adequate levels of youth MHL significantly improved one's ability to recognize own mental health status as well as provide peer support. Practical considerations such as designing more gender and culturally specific youth MHL programs are proposed.

## Introduction

Mental health is a fundamental component of the overall health of an individual. In recent decades, the concept of mental health and wellbeing has been a main topic of conversation due to the rise in mental illness burden in Australia. According to data from the Australian Bureau of Statistics (2018), one in five (20%) Australians had a mental illness, increasing from 18%, in 2014–2015. Youth mental health and wellbeing is a tremendous concern due to the rise in the incidence of mental illness, with almost half of the population with mental illness experiencing their first episode by the age of 18 (1, 2). Adolescence is a crucial time of an individual's life with many physical, emotional, and social changes, making them more vulnerable to mental illness. Psychological distress at a young age can have detrimental effects and lead to; high-risk behaviors, academic failures, unemployment, poor sexual health, self-harm, and premature mortality (3). A 2017 youth survey by Mission Australia (4) demonstrated that mental health issues (33.7%) and alcohol and other drugs (32.0%) were two of the main issues recorded among the youth participants.

The Australian population consists of many culturally, and linguistically diverse (CALD) populations born overseas or have parents born overseas who speak different languages (5). According to 2016 census data, CALD peoples made up 45% of the Australian population (6). The mental health needs of CALD populations are complex due to resettlement stressors, and strong cultural and religious beliefs associated with mental illness (2). Furthermore, CALD communities are more likely to have lower Mental Health Literacy and help-seeking behaviors than Australian-born communities (7).

Anxiety and mood disorders are the main contributors to the mental illness burden among Australian youth (5). Although early intervention is key in reducing the impacts of mental illness, evidence indicates that most youth are hesitant in seeking help due to confidentiality concerns, shame in disclosing their mental health issues, limited knowledge of mental health and wellbeing, and limited knowledge and capacity in accessing the available services (1).

Mental health literacy (MHL) was first defined as knowledge regarding mental health issues that contribute to early recognition, management, and treatment of mental illness (8). Jorm (9) expanded on the initial definition by expanding MHL to include; knowing how to prevent mental illness, the ability to recognize signs and symptoms of mental illness at an early stage, awareness of help-seeking options and available treatments, awareness of self-help strategies, and mental health first aid skills that help and support others affected by mental illness. MHL can also aid in improved help-seeking behaviors, which can reduce or halt illness progression, improve quality of life and improve levels of developmental goals among the youth.

Although improving physical health and wellbeing is popular, improving mental health and wellbeing is still perceived as novel. It is established that improved levels of mental health promotes better health and wellbeing (10). Data indicates recent improvement in MHL levels among the youth since the initial research and the introduction of MHL by Jorm et al. (8). However, the prevalence rates of youth mental illness in the latest national surveys suggest a need for more research and strategies to enhance the levels of MHL among the youth of Australia (11). This study aims to examine evidence of youth MHL, impact of MHL on mental health status and current interventions to improve and enhance MHL. The study focuses on cultural, social and environmental determinants.

## Social determinants and mental health literacy

### Gender/sex and sexual preference disparities

Gender/sex has an influence on MHL levels among youth. Studies show that females are more likely to have increased levels of MHL regarding generalized anxiety disorders compared to males due to high prevalence (12–14). Similarly, a cross-sectional study with 1,207 Australian youth participants demonstrated that females are significantly more likely to identify depression than males (15). Young females have also consistently reported high rates of psychological distress compared to young males (16).

Hegemonic masculine ideals discourage vulnerability, weakness, or emotional expression (17). While expressions of masculinity are diverse, adolescent males with perceptions of traditional masculine norms are more likely to have a greater risk of mental health issues risks and reduced help-seeking behaviors (18). Similarly, the rates of psychological distress among adolescent males have recently risen substantially (29%) compared to the rates in 2018 (20%) (19). Improving MHL in young men may help in providing more knowledge and awareness of mental illness, benefiting mental health outcomes.

Lesbian, Gay, Bisexual, Transgender, Queer, Intersex and Asexual (LGBTQIA+) youth face detrimental health challenges and are extremely vulnerable to mental health problems in the heteronormative society. A systematic review by Wilson and Cariola (20) analyzed 34 qualitative studies and found that being victims of marginalization, isolation and rejection and phobic behavior is linked with negative mental health outcomes such as self-harm, depression, and suicidality. The authors also found a need for more supportive schools and social environments for LGBTQI+ youth. While studies related to MHL levels of sex and gender minority youth are scarce, it can be established that more research needs to focus on providing necessary support and resources in improving MHL.

### Geographic locality

Geographic location can factor toward an individuals health in many ways. For example, the Prevalence of mental illness is higher among the youth living in rural areas than youth living in metropolitan areas and higher levels of barriers to necessary treatment (1). A systematic review by Gulliver et al. (21) reported that, out of the 13 qualitative studies, four qualitative studies showed a lack of accessibility due to time, transport and costs as a prominent barrier in seeking the necessary professional help among the youth living in rural areas.

Marshall and Dunstan (22) conducted a vignette short film based study with 122 participants (49 males and 73 females) from the age range of 12–18 years and examined that rural adolescents show low levels of MHL compared to metropolitan adolescents. Conversely, the study reported that the recognition of mental illness such as depression was similar among rural and metropolitan adolescents. The authors also found that rural adolescents showed low awareness of the available mental health professional help compared to metropolitan adolescents. Moreover, in association with gender/sex disparities, Marshall and Dunstan (22) also found that there is a gender difference exist concerning MHLlevels among rural adolescents compared to metropolitan adolescents. The authors also found that MHLlevels of rural adolescent males are less than rural adolescent females.

### Knowledge is power

Promoting MHLamong parents and educators are an indirect way of improving MHLamong the youth population of Australia. Youth with parents with mental illness are at more risk of developing mental illness than youth who have parents without mental illness (23). Being the caregiver of a parent with mental illness can impede the development of the child. While not all children have adverse effects, some young people build better resilience, better understanding and knowledge about mental illness. Improving the MHLof the youth of a parent with mental illness is beneficial in building and improving positive coping strategies for better mental health (24). Subsequently, youth with adequate levels of MHLare more likely to have better mental health knowledge, confidence in recognizing their mental health status and symptoms, which promotes better mental health outcomes (10).

Furthermore, Johnco and Rapee (25) found that parental depression literacy strongly influenced their parental response to an adolescent with depressive symptoms. The authors also found that poorer depression literacy of parents was associated with low support, more overprotective and distressed/withdrawn response. In contrast, parents with adequate depression literacy are more likely to support and promote positive responses toward the depressed youth. Moreover, improving parents' depression literacy was also beneficial toward the parents with managing their depressive symptoms (25).

Schools and educators are a great way of providing adolescents with a safe, supportive environment to improve their MHLas they spend most of their time in school. For example, a cluster randomized trial by Jorm et al. (26) provided mental health first aid training to middle school teachers in seven schools in South Australia to evaluate the mental health knowledge and their ability to help students with their mental health problems. After 6 months of follow-up, the authors found that the training increased teachers' mental health knowledge, changed their beliefs, and improved their ability to help students with mental health problems; however, the effects were not significant. An indirect effect noted in the study was that students reported receiving more mental health information from their teachers due to the training. Conversely, Uribe Guajardo et al. (2) found that MHFA trained teachers and responsible adults showed significant improvements in knowledge regarding youth mental health problems (*p* < 0.01) and significantly increased levels of confidence in helping youth with mental health problems (*p* < 0.001). Thus, it demonstrates teachers' key role in improving mental health knowledge to adolescents in school settings.

## Mental health literacy of culturally and linguistically diverse youth

### Culture

Culture influences many aspects of mental health, mental illness and the ways people access help toward improving their mental health status. While Australia is a major multicultural hub, other countries such as United States, South Korea and Europe are also some of the popular migrant destinations among migrant communities. Culturally and linguistically diverse (CALD) youth are more vulnerable to mental illness (2). Culture and religion surround most values and beliefs of CALD communities. Although there are generally positive influence of such culturally and religiously rich contexts, in most situations, changes that occur due to transitioning from a strong cultural influence to new social structure may result in varied negative impacts toward levels of mental health among the youth populations.

Furthermore, shame and stigma strongly influence the attitudes and behaviors of being closed off regarding mental ill-health and psychological distress and not seeking necessary help among CALD youth (27). Consequently, the patterns of shame and stigma create added stress for young people going through psychological distress. Thus, increased levels of MHLmay help CALD youth navigate their perceptions and beliefs which aids in better mental health.

### Barriers in accessing services

Increasing levels of CALD communities in Australia necessitates culturally relevant health services. While this is not the case for all care providers, CALD communities are more selective in picking their health care provider due to this reason. Additionally, Cultural competency among healthcare providers facilitates help-seeking behaviors among the youth (27). In addition, Cross and Singh (28) examined the importance of cross-cultural communication among CALD clients and Mental health care providers. The authors found that being culturally sensitive and having good cultural communication aids in gaining the client's trust and building rapport.

A qualitative study by Valibhoy et al. (29) found that many young refugees are unaware of the available services. They believe that the threshold for entering the services is severe mental illness. Additionally, cultural mistrust of the western mental health services, especially with refugee communities with complex mental health needs, deters from seeking professional mental health help (27). Furthermore, in a systematic review by Gulliver et al. (21), out of 13 qualitative studies, five qualitative studies showed negative perceptions toward mental health professionals, with issues relating to confidentiality and trust (“I am afraid counselor will pass information about me to other people”) and a preference of self-reliance (“prefer to handle myself”) over seeking professional help among the youth population.

A qualitative study by Colucci et al. (30) found that service providers understanding of ethnocultural backgrounds, migration pathways, possible trauma, and considerations of cultural interpretations of health and illnesses are instrumental in engaging refugee youth in mental health services. Moreover, implementing measure to improve MHL among the refugee youth were more likely to facilitate better access and engagement with mental health services.

Refugee youth participants in a mixed-method study by Posselt et al. (31) reported a need for more activities to promote mental health awareness and knowledge among their communities which will aid in better MHL among them. In Addition, the service providers interviewed in the study acknowledged the need. They added that limited funding to such measures is a barrier in implementing mental health awareness programs in the area. Consequently, Reducing the mental health service disengagement and facilitating appropriate measures to improve MHL is crucial in improving mental health outcomes among the CALD youth (18).

## Help seeking attitudes, behaviors, and youth mental health literacy

### Behaviors toward help-seeking

#### Alcohol and substance abuse

Horyniak et al. (32) conducted a qualitative study with young African migrant and refugee participants showed that injecting drug use (IDU) is common among them, even though it is highly stigmatized and hidden from their family and friends, they are more likely to engage in IDU. The authors demonstrate that factors such as traumatic experiences related to migration and resettlement are prominent in the susceptibility to IDU among the African migrant and refugee participants in the study. There is also a gender difference observed where, Males were significantly more likely to use alcohol as a method of dealing with depression and compared to females (15).

Consistently, McCann et al. (27) found that lack of MHL is a barrier to help-seeking behaviors. Authors also found that migrant youth are reluctant to seek help for mental health problems and substance abuse problems due to stigma-related reasons and avoid bringing shame upon their families and communities. While the authors claim a lack of generalisability in the study, the authors conclude that the need for culturally competent care is evident. Similarly, Posselt et al. (31) showed that culturally responsive complex treatment is vital in treating CALD communities with Comorbid conditions of mental illness and alcohol and other drug problems.

### Attitudes toward help-seeking

According to Wright et al. (33), while the ability to recognize and label a mental disorder may not be sufficient in determining the appropriate help, it may indicate good mental health literacy. Ratnayake and Hyde (14) investigated the relationship between MHL and help-seeking intentions among senior high school students. The Melbourne based study involved senior high school student participants (*n* = 32, 22 females and 10 males). The authors found no indication of a relationship between MHL and help-seeking intentions in the study. The authors claim that this could be due to the study's small sample size and the homogeneity of the participants or inclusion bias. Conversely, some studies found increased MHL levels resulting in improved help-seeking intentions among the youth (34, 35).

Previously identified themes of gender disparities linked with variations to help-seeking attitudes among the youth. Particularly, Males are more likely to have reduced help-seeking attitudes than females (18). Moreover, even though Ratnayake and Hyde (14) found no evidence of gender difference and help-seeking, the authors found that young males were more likely to seek professional mental health help from a doctor or a general practitioner (GP) than young females.

## Effectiveness of youth mental health literacy intervention programs

A cluster-randomized crossover trial by Hart et al. (34) compared students' (Randomized *n* = 1,942) outcomes of teen mental health first aid (tMHFA) training and physical first aid (PFA) training programs. It evaluated that tMHFA trained students showed significantly greater confidence levels to support peers, while PFA trained students felt less confidence in helping a peer with mental distress. Similarly, Uribe Guajardo et al. (2) conducted a CALD focused tMHFA and youth mental health first aid training (YMHFA) and found significantly increased levels of helpful intentions after training (*p* < 0.01) and maintained levels of helpful intentions after 3 months follow up (*p* < 0.05). Furthermore, in relation to the earlier discussed theme of low MHL levels in rural communities, Anderson and Pierce (36) found that rural-community MHL programs (MHFA programs) have increased the MHL levels, which aids in increased confidence in helping other with mental illness.

Riebschleger et al. (24) explored the effectiveness of a MHL program called Youth Education and Support (YES) program among school students in the USA. The authors found that the youth participants (*n* = 46) reported a significant improvement in their MHL levels from the pre- to post-program. Similarly, Marinucci et al. (37) aim to implement the YES program for Australian secondary school students to explore the effectiveness in the Australian context.

A cluster randomized control trial by Perry et al. (38) examined a school-based intervention program called “HeadStrong” on secondary school students (*n* = 380) and found that depression literacy levels have significantly improved post-program. However, the program did not have a significant impact on the help-seeking attitudes of the participants. The authors claimed that this could be due to the insufficient duration of the “HeadStrong” program.

### Mental health literacy for improving mental health outcomes due to the recent COVID-19 pandemic

No doubt the enforced lockdown restrictions due to COVID-19 have resulted in strong adverse effects on the mental health and wellbeing of the youth population of Australia. Such effects are mostly related to low levels of social connectedness, the uncertainty of the future, disruptions toward young people's education and employment. Such disruption of the norms may have negatively influenced the level of MHL and support these young people would have otherwise received. According to Headspace mental health survey (2020), at the early stages of the COVID-19 pandemic, the results indicated that 74% of participants reported that their mental health has declined due to COVID-19 outbreak in Australia. The survey results also indicate that 86% of participants had negative impacts to their mood, wellbeing, and sleep due to the COVID-19 pandemic. Subsequently, the survey also demonstrated that young people have been engaging in many self-care and coping strategies such as talking to friends, significant other, family and engaging in hobbies to maintain and improve their mental health and wellbeing. Similarly, Li et al. (39) found that adolescents with pre-existing mental illness experienced heightened levels of psychological distress and greater levels of loneliness and sleep disturbances due to the COVID-19 pandemic compared to adolescents without a pre-existing mental illness. It is observed that adequate levels of MHL help engage in positive self-care and coping strategies (24).

The young population of Australia have experienced adverse impacts on their mental health during the early months of the pandemic (40). The above prospective study also examined increased levels of depression and anxiety symptoms since the COVID-19 pandemic started. However, the study results show no significant changes in help-seeking from mental health professionals due to the enforced COVID restrictions. Conversely, Headspace mental health survey (2020) results indicate that the participants who engaged in help-seeking from health professionals found the help to be beneficial in managing their mental health. Thus, it can be established that improving MHL among the youth may help reduce the long-term impacts to their mental health due to COVID-19 and the enforced lockdowns.

## Discussion

The current study sought to provide a perspective on youth mental health literacy, factors contributing to MHL and its implications on mental health. The influence of social determinants on youth MHL demonstrates a gap between MHL levels among rural and metropolitan youth and its adverse effect on their mental health. The findings also touch on the gender disparities that have a greater effect on MHL levels of Australian youth. The authors established experience with young people and factors that impact their resilience and development, has facilitated extensive understanding of the scope and challenges associated with engaging youth in mental wellbeing. While there are direct ways of attaining and improving youth mental health literacy, there is no doubt that empowering communities including improving parents and educators' MHL can significantly improve youth mental health knowledge.

We also highlight here the importance of help-seeking behavior to prevent mental health issues and for early interventions. Research indicates that low MHL levels contribute to low help-seeking behaviors and attitudes. Additionally, we are well aware that youth in particular are more likely to develop unhealthy behaviors such as alcohol and substance use as coping strategies to deal with mental illness. This further highlights the importance of MHL and the provision of the necessary knowledge and understanding to improve help-seeking behaviors and attitudes.

MHL programs are crucial in improving mental health literacy. However, it is now well established that MHL programs cannot be developed as one-size-fits-all. For instance, MHL programs for adults should not be the same as those for adolescents. MHL helps build human developmental resilience. Programs for adolescents should mainly be tailored for adolescents' developmental levels and should be delivered mainly in school environments (41). Moreover, CALD communities have varied mental health needs which is best approached with culturally appropriate programs to improve mental health literacy, help-seeking behaviors among the CALD youth. Mental health service delivery needs to be culturally appropriate. In addition, mental health service providers need to count the cultural competency required to provide efficient and equitable services to the CALD communities (28). Furthermore, countries such as United States, European countries and South Korea are some of the well-known migrant destinations consisting of many migrant youths. It is more likely that they face similar challenges in improving their mental health literacy. Therefore, study findings undoubtedly can be generalized to indigenous and migrant youth living in other developed countries.

Most reviewed interventions in the current study demonstrated that the short duration of interventions can impact the sustainability of the long-term improvements in MHL (2, 18). To develop better youth MHL intervention programs, more research is needed to assess the effectiveness of school-based youth MHL programs (42) as well as a longer follow up period to evaluate the effects of MHL programs on Australian youth (43). Successful MHL interventions should take into consideration cultural, social, political, and economic factors, and, should take the bottom-up approach when working with the community and empowering its members and leadership. This will ensure better engagement and more sustainable plans. Programs developers and designers also need to construct gender-specific, culturally responsive programs for better outcomes (44, 45).

## Conclusion

MHL aids in understanding one's mental health status, acquiring knowledge of the available services, improving help-seeking behaviors and attitudes, and having the mental health first aid skill confidence in supporting a peer with mental health problems. In summary of the results, this perspective study clearly illustrates the MHL levels of Australian youth, the factors that contribute to improving MHL and its impacts on mental health. The study also demonstrates the current Australian MHL programs and their outcomes on youth mental health. However, the study raises the questions of the long-term efficacy of the programs on maintaining MHL among the youth and the need for more effective, engaging and community-based specific MHL programs for multicultural and minority groups.

More research including large longitudinal studies are required to determine causality and the strengths of association between the different themes discussed in the current study. In addition, a scoping or systematic review may be required for more robust evidence related to MHL and impacts on mental health among Australian youth. Conclusively, policymakers and health care providers should consider employing these findings as a foundation to construct and develop programs for improving MHL among Australian youth.

## Data availability statement

Publicly available data were analyzed in this study. This is a review/perspective where secondary data was gathered then analyzed and evidence summarized.

## Author contributions

HB and FH designed the perspective, developed the protocol, and identified study list for the inclusion in this perspective. FH and BA reviewed the structure and the design and recommended the structure. HB, BA, and FH reviewed final version prior to submission. All authors contributed to the article and approved the submitted version.
